# A rapid and high-throughput microplate spectrophotometric method for field measurement of nitrate in seawater and freshwater

**DOI:** 10.1038/srep20165

**Published:** 2016-02-01

**Authors:** Jiapeng Wu, Yiguo Hong, Fengjie Guan, Yan Wang, Yehui Tan, Weizhong Yue, Meilin Wu, Liying Bin, Jiaping Wang, Jiali Wen

**Affiliations:** 1 State Key Laboratory of Tropical Oceanography (LTO), South China Sea Institute of Oceanology, Chinese Academy of Sciences, Guangzhou, 510301, P. R. China; 2 Key Laboratory of Tropical Marine Bio-resources and Ecology, South China Sea Institute of Oceanology, Chinese Academy of Sciences, Guangzhou, 510301, P. R. China; 3 University of Chinese Academy of Sciences, Beijing, 100049, P. R. China; 4 School of Environmental Science and Engineering, Guangdong University of Technology, Guangzhou, 510090, P. R. China

## Abstract

The well-known zinc-cadmium reduction method is frequently used for determination of nitrate. However, this method is seldom to be applied on field research of nitrate due to the long time consuming and large sample volume demand. Here, we reported a modified zinc-cadmium reduction method (MZCRM) for measurement of nitrate at natural-abundance level in both seawater and freshwater. The main improvements of MZCRM include using small volume disposable tubes for reaction, a vortex apparatus for shaking to increase reduction rate, and a microplate reader for high-throughput spectrophotometric measurements. Considering salt effect, two salinity sections (5~10 psu and 20~35 psu) were set up for more accurate determination of nitrate in low and high salinity condition respectively. Under optimized experimental conditions, the reduction rates were stabilized on 72% and 63% on the salinity of 5 and 20 psu respectively. The lowest detection limit for nitrate was 0.5 μM and was linear up to 100 μM (RSDs was 4.8%). Environmental samples assay demonstrated that MZCRM was well consistent with conventional zinc-cadmium reduction method. In total, this modified method improved accuracy and efficiency of operations greatly, and would be realized a rapid and high-throughput determination of nitrate in field analysis of nitrate with low cost.

Nitrate, the most oxidized form of inorganic nitrogen in aquatic environments, is known as an important bioavailable nitrogen source and a major nutrient to support primary productivity in the seawater and freshwater ecosystem. Over most of the surface ocean, the concentration of NO_3_^−^ is below detection level, so it is often become a limit factor of primary productivity^1^,^2^. Driven by biological pump and complex microbial transformations, NO_3_^−^ increases very rapidly with depth in the ocean, and the average concentration in the deep ocean water is ranged from 20–30 μM^3^. With the upwelling or dynamic mixing, the nitrate stored in the deep ocean is transported to the surface zone for biological productivity process^4^. In contrast, the nitrate content in estuarine waters, is rising at an alarming rate, mainly due to the anthropogenic activity, such as urban and agricultural runoff, insufficient treatment of domestic and industrial wastewaters^5^,^6^. Overloading nitrogen in the coastal zones has led to critical environmental problems, for example eutrophication^7^, hypoxia expansion^8^,^9^ and nitrous oxide emissions^10^.

In order to investigate the ecological and environmental effect of nitrate, it is essential to determine the nitrate concentration in the seawater or freshwater accurately. Currently, numerous methods for nitrate detection have been reported, including spectrophotometric, fluorescent, chemiluminescent and chromatographic assays. Highly sensitive techniques are usually depended on the expensive and specialized large-scale equipment, including chemiluminiscence^11^, gas chromatography-mass spectrometry^12^, ion-chromatography^13^, capillary electrophoresis electrophoresis^14^ and potentiometry^15^ and so on. Although the nitrate can be quantified at very low concentration with these methods, it might be difficult to apply them directly in the field research for real-time determination. Compared to these expensive techniques, spectrophotometric methods are by far the most widely used for nitrate determination based on the utilization of metallic granules, such as zinc^16^,^17^, cadmium^18^,^19^, vanadium^20^, hydrazine-copper^21^,^22^, and copperised cadmium^23^. Under the singe or combined action of metallic granules, nitrate can be reduced to nitrite for further detection based on the formation of a pink-colored azo dye derived from diazotizing nitrite with sulfanilamide and coupling with N-1-naphthylethylenediamine dihydrochloride^24^,^25^. Among them, the well-known zinc-cadmium reduction method is most widely used for nitrate determination in seawater due to its low cost and easy operation without using expensive instruments. However, zinc-cadmium reduction method has some disadvantages to limit its application in the field research. First, the reduction rate of nitrate is not stable as the salinity effect. Under different salinity condition, the reduction rates vary significantly, leading to a possible large measurement error. Second, it is a time-consumption operation for the detection with a spectrophotometer one by one. At last, the classic zinc-cadmium reduction method needs relatively lager volumes of water samples (usually 25 mL), so it would be not sufficient to analyze NO_x_^−^ by this method when the volume of water sample was lower than 25 mL.

For overcoming these deficiencies, we developed a rapid and high-throughput microplate spectrophotometric method for determination of nitrate with small volumes by modified previous zinc-cadmium reduction method in this study. This modified method not only improved the reduction rate and stability of the nitrate to nitrite significantly, but also simplified operation procedures and realized high-throughput detection of nitrate with a microplate spectrophotometric method.

## Results

### Optimization of the reaction conditions of MZCRM

Based on a univariate experimental design, the three possible affecting factors of salinity, reduction time and size of zinc roll were analyzed to optimize the reaction condition of the method.

#### Effect of salinity on the nitrate reduction rate

**Figure 1:**
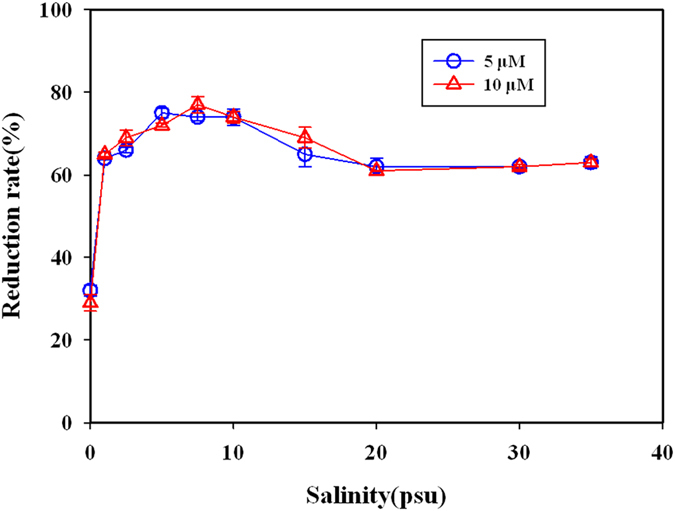
Effect of salinity on nitrate reduction rate. Artificial seawater of varying salinity (0–35 psu) containing 5 μM and 10 μM nitrate were used as standards, respectively. Error bars represent standard deviation from a triplicate sampling analysis.

The effect of salinity on nitrate reduction rate was showed in Fig. 1. The reduction rate of nitrate of both at 5 μM and 10 μM had similar shifting trends. When the salinity was zero, nitrate reduction rate was only 30%. However, the nitrate reduction rate increased rapidly with a little increase of salinity. When the salinity was 2.5 psu, reduction rate have been up to 62%. Then, the reduction rate increased slightly and reached on the maximum value of 78% at salinity of 8 psu. Next, the reduction rate began to decline slowly to 65% until the salinity was up to 20 psu. When the salinity was between 20 and 35 psu, the nitrate reduction rate remained in a stable range of 63%.

The variation trend of nitrate reduction rate indicated that the salinity have significant effect on the nitrate reduction. Under the condition of lower salinity from 5–10 psu, the reduction rate remained relatively stable and higher. In contrast, the reduction rate remained relatively stable but lower under the condition of higher salinity of 20–35 psu. In order to improve the precision of nitrate detection in the water samples, two-section methods was suggested to determine the nitrate concentration.

#### Effect of reduction time

**Figure 2:**
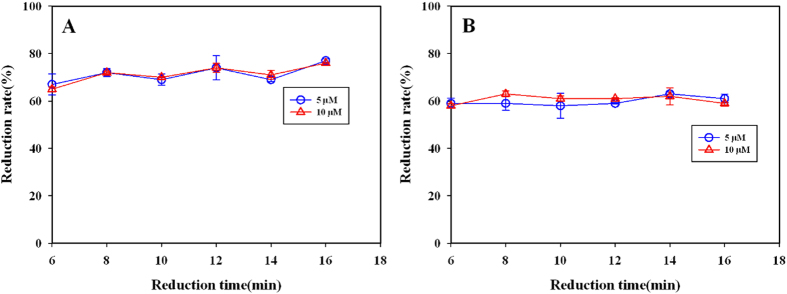
Effect of reduction time (min) on nitrate reduction rate. Both 5 μM and 10 μM nitrate were used at salinity of 5 psu (**A**) and salinity of 20 psu (**B**).

The effect of reduction time on the reduction rate was shown in Fig. 2. From 6 min to 8 min, the reduction rate had a slight increasing under the both conditions of salinity of 5 and 20 psu. Then the reduction rate remained stable in a range from 60–63% over 8 min, suggesting the reduction rate has reached maximum at 8 min. No significant difference was observed when nitrate concentration was used as 5 μM and 10 μM in the reaction system. At last, 10 min was selected as optimal reduction time for further analysis.

#### Effect of the size of zinc roll on the nitrate reduction rate

**Figure 3:**
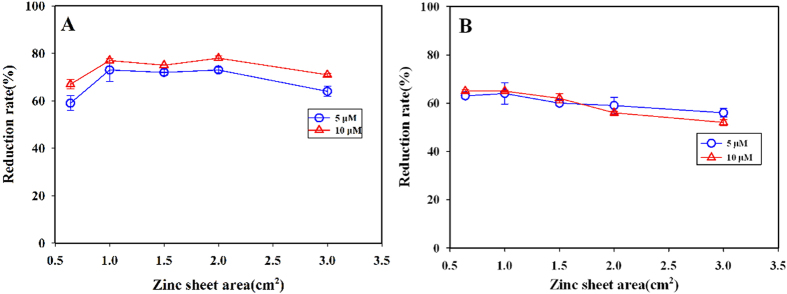
Effect of zinc sheet area (cm^2^) on nitrate reduction. Both 5 μM and 10 μM nitrate were used at salinity of 5 psu (**A**) and salinity of 20 psu (**B**) in these experiments.

At two different salinity levels of 5 and 20 psu, the effects of the size of zinc roll on the nitrate reduction rate were remarkable different. When the salinity was 5 psu, the reduction rate was increase rapidly with the increasing of the zinc roll size from 0.64–1 cm^2^, reached a maximum at 1 cm^2^, and began to decrease slightly when the size was over 3 cm^2^ (Fig. 3A). When the salinity was 20 psu, the reduction rate remained stable with the size of zinc roll from 0.64–1 cm^2^, and then decrease slowly with increasing areas of zinc roll from 1–3 cm^2^(Fig. 3B). The minimum reduction rate occurs at the area of 3 cm^2^, which is blow 60%. Combined the results at two different salinity, the size of 1.5 cm^2^ was chosen as an optimal catalyst area of zinc roll.

### Stability and detection limit

**Figure 4:**
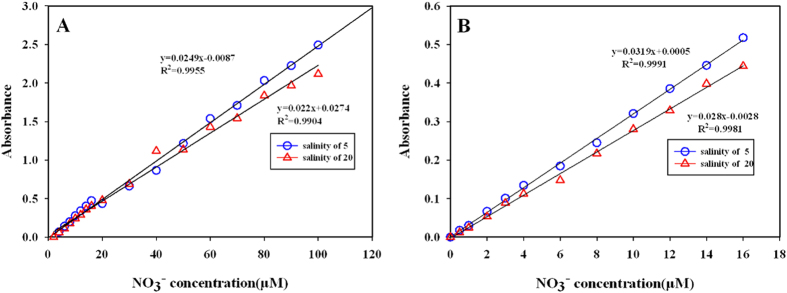
Standard calibration curve under optimal condition. (**A**) 0.5–100 μM, (**B**) 0.5–16 μM.

Under the condition of optimized reaction conditions, the stability and detection limit of nitrate with this method were defined. Our experimental data showed that the lowest detection limit was 0.5 μM and the highest detection limit was up to 100 μM, which covered most concentration range of nitrate in the environmental waters. Precise linear regressions between absorbance value and concentration of nitrate below 16 μM in both matrix salinity of 5 and 20 psu were showed in Fig. 4B. The linear regression equations were listed as following (eqs 1 and 2):


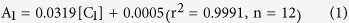



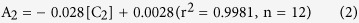


Where A_1_ and A_2_ were the absorbance, C_1_ and C_2_ were the concentration of nitrate in matrix salinity of 5 and 20 psu, respectively. The different slopes between two standard calibration curves indicated that there were different reduction rates at salinity of 5 (72%) and 20 psu (63%), which was consistent with the result in the section of effect of salinity on the nitrate reduction rate.

When the concentration of nitrate was extended to 100 μM, the linear regression equations between absorbance value and concentration of nitrate at salinity of 5 and 20 psu were showed in Fig. 4A. The linear regression equations were listed as following (eqs 3 and 4):


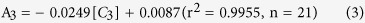



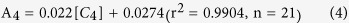


Where A_3_ and A_4_ were the absorbance, C_3_ and C_4_ were the concentration of nitrate in matrix salinity of 5 and 20 psu, respectively. The r^2^ and the slope of calibration curve in this detection range were both slightly lower than in the detection range of 16 μM, which suggested a decreased stability when the detection range was enlarged.

### Application to environmental samples assay and cross-validation

**Figure 5:**
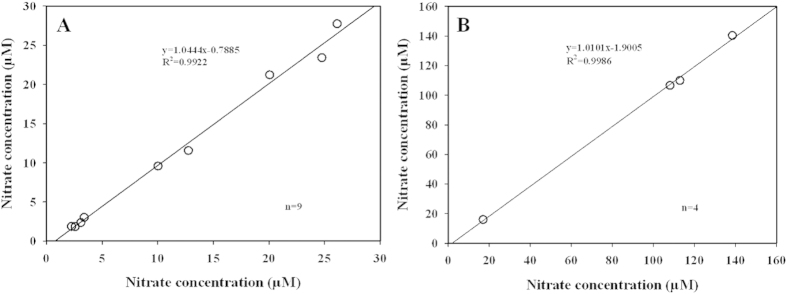
Comparison of the proposed method and reference method. (**A**) samples from South China Sea; (**B**) samples from Pearl River Estuary.

To confirm the stability and precision of our new developed method, the concentration of nitrate of environmental samples was assayed with MZCRM and classical zinc-cadmium method simultaneously. Results showed that MZCRM and classical zinc-cadmium method fitted well for water samples (Fig. 5A,B). As shown in Fig. 5A, the analysis of seawater from South China Sea using MZCRM_h_ (the standard regression of high salinity section) were highly correlated with those using classical method, with a slope of 1.0444 and a regression coefficient of 0.9922, Moreover, we determined the samples from the Pearl River Estuary with MZCRM_l_ (the standard regression of low salinity section) and classical method (adding sodium chloride to adjust salinity to exceed 25 psu) simultaneously. The results in Fig. 5B showed that data of nitrate concentrations determined using MZCRM_l_ were also matched at relatively accuracy with the data using classical method, with a slope of 1.0101 and a regression coefficient of 0.9986. The relative standard deviations (RSDs) at different nitrate concentrations ranging from 2.2–140.3 μM were in average of 4.8%, t-test showed that the result of two methods are homogenous (seawater: t = 0.81, p_1_ = 0.4433 > 0.05, n = 18; freshwater: t = 0.92, p_2_ = 0.4255 > 0.05, n = 8). These statistical analyses indicated that MZCRM and classical zinc-cadmium method should be no significant difference in the nitrate concentrations measurement.

We then validated the error analysis between the low (5–10 psu) and high (20–35 psu) section method using two different salinity samples (Table 1). The results showed that there was large error (RSDs: 13%) between the data using MZCRM_l_ (matrix salinity of 5 psu and calibration curve of 5 psu) and those using mismatch method (matrix salinity of 5 psu and calibration curve of 20 psu) to determine the standard sample of low salinity. Additionally, there was also a large error (RSDs: 14%) between the data using MZCRM_h_ (matrix salinity of 20 psu and calibration curve of 20 psu) and those using mismatch method (matrix salinity of 20 psu and calibration curve of 5 psu) to determine the standard sample of high salinity. So the determination of nitrate concentration should be using MZCRM_l_ under the condition of low salinity and the determination of nitrate concentration should be using MZCRM_h_ under the condition of high salinity. In order to improve the precision of nitrate detection in the water samples, two-section methods (salinity of 5~10 psu and salinity of 20~35 psu) was essential to determine the low salinity and high salinity sample, respectively.

## Discussion

### Salt effect

In our study, the reduction rates of converting nitrate to nitrite were different under different salinity, ranged from 30%–78%, indicating salinity should be a crucial factor for nitrate reduction and single salinity of matrix can not be used to fulfill stability of detection. When the salinity is zero, nitrate reduction rate was relatively low. This may be resulted from the low adhesion force between cadmium ion and the surface of zinc sheet under the lower salinity (salinity < 2 psu), because the film of Zn_2_(OH)_2_CO_3_ attached to the surface of zinc will easily be destroyed by brine solution or acid solution^26^. Without the coactions of zinc and cadmium, the nitrate could not be sufficiently converted to nitrite. However, a rapid rise of reduction rate can be observed with a little increase of salinity. When the salinity was only 2.5 psu, reduction rate have been up to 62%. This supported that ionic strength of matrix destroyed the oxidation film of zinc, thus zinc can be exposed to cadmium and reacted as microbattery. The reduction rate increased slightly and reached on the maximum value of 78% at salinity of 8 psu. Similar findings previously by Zhang and Fischer^27^ were reported that nitrate reduction rate increased approximately 2 folds from salinity of 2 psu to salinity 5 psu and reached a maximum value at a salinity of 10 psu. And this may be due to the fact that the ionic strength of water medium has a significant effect on the zinc-cadmium reduction process, which not only improved the solubility of CO_2_ in water but also accelerated the hydrolysis of H^+^. In addition, increasing of the electrical conductivity and formation of zinc-cadmium microbattery caused by the ion effect will further improve the efficiency of reduction^26^,^28^.

Compared to the nitrate reduction rate on the salinity of 5–10 psu, a slight decrease of that between salinity of 10–20 psu was observed. Several alternative explanations have existed for explaining why reduction rates decreased along with the salinity increasing. First and most, higher salinity can result in a higher ionic strength in the sample solution, and based on the Debye-Huckel equation, the activity coefficients for ions in the sample solution decrease as the ionic strength increases^29^,^30^. Moreover, increasing the chloride concentration produced a decrease in brucine-nitrate color development^31^. In the previous studies, multiple kinds of methods have been proposed for the determination of nitrate in ocean, estuarine and freshwaters, but the reduction process of converting nitrate to nitrite were significantly influenced by the existence of chloride in most of these methods^32^.

It should be noted that the reduction rates remained stable in salinity between 20–35 psu. However, little attention has been focused on this stable section of salinity. It was proposed in the Specification for oceanographic survey^33^ that 0.5 g of sodium chloride should be added to each samples to adjust the salinity when the salinity of samples were lower than 25 psu. This treatment should be based on the fact that reduction rates are stable for determining nitrate under the condition of the salinity ranged from 25–35 psu. However, the specific chemical mechanism remains unknown and needs further study.

Feng *et al*.^34^ suggested that using low-nutrient seawater as a matrix for preparing the calibration standards would overcome the salt effect. In fact, adding sodium chloride to water samples to adjust salinity and using low-nutrient seawater as a matrix will all generate a great influence on the precise of nitrate detection. With the increase of matrix salinity, the reduction rates decreases from 78% – 60% (Fig. 1). It is suggesting that lower reduction rate and higher ionic strength response to lower detection limit and higher blank value. The determination sensibility will be reduced when adding sodium chloride to sample to adjust the salinity exceed 25 psu. Therefore, our proposed method of salinity of 5–10 psu to determine freshwater and salinity of 20–35 psu to determine seawater could better display the actual nitrate concentration.

### Linear dynamic range and detection limit

Accurate determination of nitrate concentrations at submicromolar concentration is essential in aquatic biogeochemical processes. In the last couple of decades, how to determine nitrate accurately and rapidly has always been a important researching issue and multiple analytical methods has been developed for this purpose^35^. Nitrate analysis is commonly based on indirect determination after reduction to nitrite by reducing metals or nitrate reductase^36^. Different method has different sensitivity and detection limit. Chemiluminescence^11^ and GCMS^12^ method have detection limit of 2 nM for nitrate but require expensive equipment, intensive labor and a large sample volume. It reported that HPLC method had a sub-nanomolar detection limit but requires purification of derivatizing reagent and skilled operators^24^. In general, using unique reverse flow injection system can fulfill the determination of nanomolar nitrate in seawater^34^.

However, none of the techniques and methods mentioned above can be able to perform a rapid, small volume, high-throughput determination of nitrate in the field research. In this study, we developed a new-type zinc-cadmium reduction method based on vital modification from classical one. Under optimal conditions, typical calibration curves of nitrate ranging from 0–16 μM were obtained at salinity of 5 and 20 psu, respectively. The high values of R^2^ (0.9991 and 0.9981 corresponding to salinity 5 and 20 psu) indicated that the reduction rates of nitrate to nitrite are stable under specific salinity condition. So, the regression equation can be used to calculate the concentration of nitrate accurately with this method. Such stability could be attributed to (1) increased the touching frequency between reagents and zinc roll with vortex apparatus, (2) introduced a two-section salinity method to determine low and high salinity samples, and (3) decreased the blank values by using pollution-free disposable tubes. It should be noted that the sensitivity and detection limit for the determination of nitrate is not big better than the classical zinc-cadmium method. This is mainly because the shorter optical distance of the well of 96-well plate than that of the cell of spectrophotometer. Whatever, this new developed small volumes method should be a convenient and rapid method for determination of nitrate concentration in seawater and freshwater without using specialized and expensive equipment, especially for the field research.

## Methods

### Instrumentation

All spectrophotometric measurements were performed with a microplate reader (Tecan Sunrise, Switzerland) equipped with sophisticated 12-channel optical module, which ensures fast measurements and high quality results. This microplate reader can process an entire 96-well plate in only 6 seconds for a single-wavelength read and in only 8 seconds for dual-wavelength read. In order to enhancing the efficiency of the nitrate reduction, 2 mL round bottom disposable tubes (Fisher Scientific LabServ, USA) were used for nitrate determination. A vortex apparatus (Scientific Industry, USA) were used for shaking of the tubes to increase touching frequency between reagents and zinc roll.

### Reagents and solutions

The water used in this study is ultrapure grade (Milli-Q) and reagents were of analytical or guaranteed grade (AR or GR). Zinc sheets of different sizes (purity of 99.99%, thickness of 0.1 mm) were prepared and were rolled into uniform cylinders. Cadmium chloride solution (20 g/L) was prepared by dissolving 20 g of Cadmium chloride (CdCl_2_·5/2 H_2_O) with 1 L ultrapure water. Sulphanilamide solution (10 g/L) was prepared by dissolving 5 g of sulphanilamide in 350 mL 14% (v/v) HCl and this solution is stable for at most two months. NEDD solution (1 g/L) was prepared by dissolving 0.5 g of N-(1-naphthyl)-ethylenediamine in 500 mL ultrapure water and it can be used until a brown discoloration occurs. Artificial seawater (salinity of 5 or 20 psu) was prepared by dissolving 5 g or 20 g of sodium chloride (GR) in 1 L pure water. Nitrate stock solutions (10 mmol/L) was prepared by dissolving 1.011 g of oven dried (110 °C, 1 h) KNO_3_ (GR) in 1 L ultrapure water and the solution is stable for at most six months. Nitrate working solutions were prepared with nitrate stock solution by dilution with pure water. Nitrite stock solutions (5 mmol/L) were prepared by dissolving 0.345 g of NaNO_2_ (GR) in 1 L ultrapure water and the solution is stable for at most two months. Nitrite working solutions were prepared with stock solution by dilution with ultrapure water. All solutions used in this study were normally stored in dark glass bottles at 4 °C.

### Standard protocol for nitrate determination

**Figure 6:**
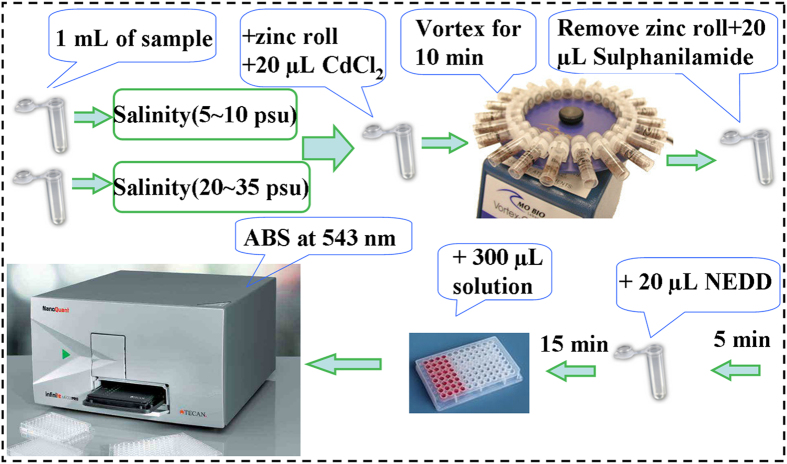
The general procedure of rapid and high-throughput microplate spectrophotometric method for determination of nitrate.

Transferred one zinc roll and 20 μL of cadmium chloride solution into a 2 mL tube with 1 mL water sample in order, and then fixed it on the Vortex-genie2 with setting the vortex speed as the speed 1^st^ (about 600 rpm) for slightly shaking 10 min at room temperature. After reaction, removed the blacken zinc roll out of the tube. Next, added 20 μL of sulphanilamide solutions into the tube and mixed it gently. After 5 min incubation, transferred 20 μL of NEDD solution into the tube and incubated for 15 min. Finally, transferred 300 μL of the liquid reactant into one of wells of 96-well microplates and measured the spectrophotometric absorbance by microplate reader at 543 nm (see Fig. 6).

### The calculation of reduction rate and concentration of nitrate

With same amount (mole) NO_3_^−^ and NO_2_^−^, the reduction rate of nitrate is calculated based on the ratio of the actual absorbance values of NO_3_^−^ after reduction to the actual absorbance values of NO_2_^−^. The reduction rate (R) can be calculated with the following equation (Eq. 5)


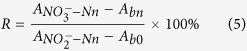


Where 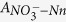 is the actual absorbance of NO_3_−N after reduction; 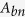 is the blank absorbance of NO_3_−N; 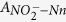 is the absorbance of NO_2_−N; 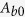 is the blank absorbance of NO_2_-N.

The actual nitrate concentration (C) of the sample is calculated as (Eq. 6):


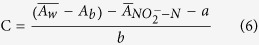


Where 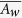 is the average of the total absorbance of sample after reduction; 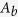 is the absorbance of blank; 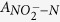 is the average of the absorbance of nitrite determined before reduction; a is the intercept of standard calibration curve and b is the slope of standard calibration curve.

### Optimization of reaction conditions and formulation of the standard calibration curve

The effect of different factors on the nitrate reduction, including salinity, reduction time and the size of zinc roll, were studied to optimize the reaction condition based on single variable method. For optimization of these factors, 5 and 10 μM standard nitrate solutions were used as testing water samples.

To study the salt effect on the reaction, the salinity was varied from 0–35 psu by diluting or evaporating the artificial seawater with interval of 5 psu. The zinc rolls used for testing salinity effect were used with uniform size of 1.5 cm^2^. The detail operation was according to the above general procedure. The reduction rates of nitrate were calculated based on the measured absorbance values under condition of different salinity.

To investigate the effect of reduction time to the reduction of nitrate, 1 mL testing water samples (with salinity of 5 and 20 psu respectively) and 20 μL of cadmium chloride solution were transferred into 2 mL tubes, followed by adding 1.5 cm^2^ zinc roll. The tubes were shook for different time varied from 6–16 min. The reduction rate was calculated with the measured absorbance in different reduction time.

The effect of the size of zinc roll on the reduction of nitrate was studied by varying the size of zinc roll from 0.64–3 cm^2^. 1 mL testing water samples (with salinity of 5 and 20 psu respectively) and 20 μL of cadmium chloride solution were transferred into 2 mL tubes, followed by adding different sizes of zinc roll. The tubes were shook for 10 min according to the general procedure. The reduction rate was calculated based on the measured absorbance in different sizes of zinc roll.

With optimized reaction conditions, the standard calibration curve was formulated at the salinity of 5 and 20 psu. The nitrate standards are prepared by dilution with nitrate-free artificial sea water to the following concentrations: 0, 0.5, 1, 2, 3, 4, 6, 8, 10, 12, 14, 16, 20, 30, 40, 50, 60, 70, 80, 90 and 100 μM nitrate-N, respectively. The formulation of the standard calibration curve was carried out by the general procedure.

### Application to environmental samples assay and cross-validation

Estuarine water samples were collected along a salinity gradient (salinity of 0–15 psu) in the Pearl River Estuary in March 2015. Seawater was collected from nine different depths (25, 50, 75, 100, 200, 300, 500, 1000 and 1400 m) using a CTD carousel water sampler in the Southeast Asia Time-Series Study station (SEATS) (21.5°N, 118°E) at South China Sea during an R/V Shiyan3 cruise in October 2014. All of the water samples were filtered through Polycarbonate microporous membrane filter with a pore size of 0.22 μm (Millipore) and stored frozen at −20 °C until analysis. In order to test the stability and accuracy of this new developed method, the seawater and estuarine water samples were measured with both new and conventional method at same time, and then compared their difference with statistical analysis.

## Conclusion

A rapid and high-throughput microplate spectrophotometric method for the sequential determination of nitrate in a small volume system was established. Based on the study of effects of salinity, reduction time and zinc roll size to the nitrate reduction, an optimal reaction condition was designed to detect concentration of nitrate in seawater and freshwater. The new developed method had the advantages of low sample consumption (<1 mL), low blank value, high accuracy (RSDs: 4.8%), low determination limit (0.5 μM), wide detection range (from 0.5 μM to 100 μM) and high efficiency (>100 samples h^−1^). The cross-validation analysis demonstrated that the new developed method was well agreement with classical zinc-cadmium methods (p_1_ = 0.4433, p_2_ = 0.4255). The method is promising to be developed an alternative for the sequential determination of nitrate in seawater and estuarine water on research vessel.

## Additional Information

**How to cite this article**: Wu, J. *et al*. A rapid and high-throughput microplate spectrophotometric method for field measurement of nitrate in seawater and freshwater. *Sci. Rep.* **6**, 20165; doi: 10.1038/srep20165 (2016).

## Figures and Tables

**Table 1:** Error analysis among the low (5–10 psu) and high (20–35 psu) section method using two different salinity samples.

**Methods**	**MS 5 → CC 5 (proposed method)**	**MS 5 → CC 20 (mismatch method)**	**MS 20 → CC 20 (proposed method)**	**MS 20 → CC 5 (mismatch method)**
Nitrate concentration (μM)	2.08	2.48	2.00	1.65
Matrix salinity	5		30	
Std	0.29		0.25	
RSDs (%)	13		14	
